# Altered Dynamic Functional Connectivity in Subcortical Ischemic Vascular Disease With Cognitive Impairment

**DOI:** 10.3389/fnagi.2021.758137

**Published:** 2021-12-10

**Authors:** Yuanhang Xu, Huajie Shang, Hui Lu, Junying Zhang, Li Yao, Zhiying Long

**Affiliations:** ^1^The State Key Laboratory of Cognitive Neuroscience and Learning & IDG/McGovern Institute for Brain Research, Beijing Normal University, Beijing, China; ^2^BABRI Centre, Beijing Normal University, Beijing, China; ^3^Institute of Basic Research in Clinical Medicine, China Academy of Chinese Medical Sciences, Beijing, China; ^4^School of Artificial Intelligence, Beijing Normal University, Beijing, China

**Keywords:** resting-state fMRI, dynamic functional connectivity, subcortical ischemic vascular disease, mild cognitive impairment, brain state

## Abstract

Subcortical ischemic vascular disease (SIVD) can cause cognitive impairment and affect the static functional connectivity of resting functional magnetic resonance imaging (fMRI). Numerous previous studies have demonstrated that functional connectivities (FCs) fluctuate dynamically over time. However, little is known about the impact of cognitive impairment on brain dynamic functional connectivity (DFC) in SIVD patients with MCI. In the present study, the DFC analysis method was applied to the resting functional magnetic resonance imaging (fMRI) data of 37 SIVD controls (SIVD-Control) without cognitive impairment, 34 SIVD patients with amnestic MCI (SIVD-aMCI) and 30 SIVD patients with nonamnestic MCI (SIVD-naMCI). The results indicated that the cognitive impairment of SIVD mainly reduced the mean dwell time of State 3 with overall strong positive connections. The reduction degree of SIVD-aMCI was larger than that of SIVD-naMCI. The memory/execution function impairment of SIVD also changed the relationship between the mean dwell time of State 3 and the behavioral performance of the memory/execution task from significant to non-significant correlation. Moreover, SIVD-aMCI showed significantly lower system segregation of FC states than SIVD-Control and SIVD-naMCI. The system segregation of State 5 with overall weak connections was significantly positive correlated with the memory performance. The results may suggest that the mean dwell time of State 3 and the system segregation of State 5 may be used as important neural measures of cognitive impairments of SIVD.

## Introduction

Cerebral small vessel disease (CSVD) is a disease that causes brain damage due to changes in small vessels in the brain. Subcortical ischemic vascular disease (SIVD) is the typical representative and a more homogeneous subtype of CSVD. It is widespread among elderly individuals with asymptomatic lacunes and subcortical white matter (WM) hyperintensities (Roman et al., 2002). SIVD frequently causes cognitive impairment and dementia (Pantoni, 2010). Subcortical ischemic vascular disease patients with cognitive impairment show cognitive deficits in mental speed, language, executive functioning, visuospatial reasoning and memory (Frisoni et al., 2002; Galluzzi et al., 2005; Seo et al., 2010). However, SIVD patients could manifest unobvious or even nonexistent cognitive impairment at an early stage, and they may have a high risk of cognitive impairment in the future (Sachdev et al., 2014). Studying the neural correlates of SIVD can advance our understanding of the mechanism through which SIVD contributes to cognitive impairment.

Functional magnetic resonance imaging (fMRI) technology has been widely applied to investigate the neural mechanism changes associated with CSVD in recent years. Cerebral small vessel disease patients with cognitive impairment show activation changes in brain regions in the frontoparietal control network (FPCN) and default model network (DMN), and these changes are related to cognitive impairment (Li et al., 2012; Papma et al., 2012). Moreover, resting-state fMRI (rsfMRI) found that CSVD patients with cognitive impairment showed impaired deactivation or hyperactivation in the DMN (Aizenstein et al., 2011; Mayda et al., 2011) and reduced functional connectivity within or between nodes of the DMN, FPCN and dorsal attention network (DAN) (Sun et al., 2011; Yi et al., 2012; Schaefer et al., 2014; Zhou et al., 2015).

For patients with SIVD, altered amplitude low-frequency fluctuations (ALFF) of resting fMRI have been found in some brain regions (Liu et al., 2014), and abnormal ALFFs are dependent on specific frequency bands (Li et al., 2014). Altered functional connectivity within resting-state networks, including the sensorimotor network (SMN), posterior DMN, right FPCN and language network (LN), was observed in SIVD patients (Liu et al., 2019). Fu’s study found that Binswanger’s disease of SIVD showed increased functional network connectivity mainly between default mode and sensory regions and decreased functional network connectivity mainly within the default mode domain and related to auditory region (Fu et al., 2020). Decreased coupling between regional homogeneity (ReHo) and cerebral blood flow (CBF) was found in patients with SIVD (Liu et al., 2021). Moreover, a task fMRI study found that patients with SIVD showed altered activation in the prefrontal cortex (Li et al., 2012).

Previous rsfMRI studies of SIVD/CSVD used static functional connectivity (FC) to characterize the temporal interaction between a pair of regions during the whole scanning period. However, recent studies suggest that FC fluctuates over time, which cannot be revealed by static FC analysis (Chang and Glover, 2010; Handwerker et al., 2012). Thus, dynamic FC (DFC) was proposed to characterize the time-varying fluctuations of FC between brain regions (Preti et al., 2017). Some studies have demonstrated that neuropsychological diseases, such as autism (Zhang et al., 2016), epilepsy (Liu et al., 2017), major depressive disorder (Long et al., 2020), bipolar disorder (Nguyen et al., 2017), and schizophrenia (Guo et al., 2018), could induce changes in dynamic FC. Moreover, Fu’s study found similar and distinct functional connectivity alterations in AD and SIVD from both static and dynamic FC perspectives (Fu et al., 2019). These findings suggest that dynamic FC is important for us to understand brain dynamic functions in psychiatric disorders.

To date, the dynamic features of brain FC in SIVD patients with cognitive impairment remain unclear. This study aimed to examine how mild cognitive impairment (MCI) changed the brain dynamic properties of SIVD FC and the relation between DFC and neuropsychological performance. Time-varying FC between brain regions was estimated using the sliding window method. K-means clustering was applied to FC matrixes of all the windows to detect connectivity states that reoccurred over time and were reproducible across subjects. In this study, some SIVD patients with MCI showed prominent impairment in memory, and some showed prominent impairment in execution function without memory loss. Moreover, MCI is generally divided into two types: amnestic MCI (aMCI), with memory loss as the predominant symptom, and nonamnestic MCI (naMCI), with prominent impairments in domains other than memory (for example, language, visuospatial, executive) (DeCarli, 2003; Petersen and Morris, 2005). Based on the subtype division of MCI in previous studies, we further divided SIVD patients with MCI into two groups: SIVD patients with amnestic MCI (SIVD-aMCI) and SIVD patients with nonamnestic MCI (SIVD-naMCI) according to the different cognitive impairment symptoms. Therefore, three groups of subjects who were SIVD-aMCI, SIVD-naMCI and SIVD controls without MCI (SIVD-Control) were included in this study. We assessed the dynamic FC of rsfMRI in SIVD-aMCI, SIVD-naMCI and SIVD-Control patients. We hypothesized that the presence of aMCI and naMCI in SIVD would induce different alterations of DFCs and alteration of relationship between DFC and the neuropsychological performance compared to SIVD-Control.

## Materials and Methods

### Subjects

A total of 101 SIVD patients were recruited in the experiment, including 37 SIVD controls (SIVD-Control) without cognitive impairment and 64 SIVD patients with MCI. According to different cognitive impairment symptoms, the 64 SIVD patients with MCI were further divided into two groups: 34 SIVD patients with amnestic MCI (SIVD-aMCI) and 30 SIVD patients with nonamnestic MCI (SIVD-naMCI). All subjects gave written consent, which was set by the MRI Center of Beijing Normal University. The experiment was approved by the Institutional Review Board (IRB) of the State Key Laboratory of Cognitive Neuroscience and Learning in Beijing Normal University.

The SIVD patients were examined by two different radiologists. The two radiologists assessed the anatomical MRI scans, which contained T1-weighted, T2-weighted, fluid-attenuated inversion recovery (FLAIR) and gave nearly the same reports. Patients were excluded on the basis of dissimilar reports. The SIVD patients met the following brain imaging criteria of SIVD: (1) Binswanger-type white matter lesions: hyperintensities extending into the periventricular and deep white matter; extending caps (> 10 mm as measured parallel to the ventricle) or irregular halos (> 10 mm with broad, irregular margins and extending into deep white matter); and diffusely confluent hyperintensities (> 25 mm, irregular shape) or extensive white matter changes (diffuse hyperintensity without focal lesions); (2) lacunar cases: multiple lacunas (> 2) in the deep gray matter and at least moderate white-matter lesions; (3) absence of hemorrhages, cortical and/or territorial infarcts and watershed infarcts; signs of normal pressure hydrocephalus; and specific causes of white-matter lesions. In addition, the visual Fazekas scale was used on FLAIR images to rate the severity of white matter hyperintensities (WMHs) into mild (grade 1), moderate (grade 2) and severe (grade 3) WMHs.

Subjects who met the following criteria were excluded: (1) no completion of neuropsychological testing; (2) Hamilton depression scale score > 17, or anxiety; (3) new strokes within three months before baseline; (4) signs of large vessel disease, such as cortical and/or cortico-subcortical nonlacunar territorial infarcts and watershed infarcts or hemorrhages; and (5) leukoencephalopathy as a result of other causes, such as normal pressure hydrocephalus, multiple sclerosis, brain irradiation, and metabolic diseases.

### Image Acquisition

All MRI data were obtained from a 3T Siemens scanner at the MRI Center of Beijing Normal University. The following procedures were employed to acquire each set of MRI images. (1) T2-weighted images (TR = 5,000 ms, TE = 105 ms, slice thickness = 3 mm, flip angle = 150°, number of slices = 33) and T2-FLAIR images (TR = 9,000 ms, TE = 81 ms, slice thickness = 3 mm, flip angle = 150°, number of slices = 25) were acquired. (2) T1-weighted, sagittal 3D magnetization-prepared rapid gradient echo (MP-RAGE) sequences were acquired and covered the entire brain [176 sagittal slices, repetition time (TR) = 1,900 ms, echo time (TE) = 3.44 ms, slice thickness = 1 mm, flip angle = 9°, inversion time = 900 ms, field of view (FOV) = 256 × 256 mm^2^, acquisition matrix = 256 × 256]. (3) A T2-weighed fluid-attenuated inversion recovery (T2w-FLAIR) sequence was applied to measure WM hyperintensities with the following parameters: TR = 9,000 ms, TE = 81 ms, slice thickness = 3 mm, flip angle = 150°, number of slices = 25. (4) An echo EPI sequence (TR = 2,000 ms, TE = 30 ms, flip angle = 90°, FOV = 200 × 200 mm^2^, matrix = 64 × 64, slice thickness = 3.5 mm, interslice gap = 0.8 mm, slices = 33) was used in the experiment. During the functional image acquisition, all subjects were asked to keep still, keep their eyes closed, stay awake and not to think of anything in particular in a single run. The resting acquisition lasted for 8min, and 240 image volumes were obtained.

### Neuropsychological Assessment

All the participants underwent a battery of neuropsychological tests: the Mini-mental State Examination (MMSE), Rey-Osterrieth Complex Figure (ROCF) Test copy and recall, Trail-Making Tests (TMT) A and B, Stroop Color-Word Test (SCWT), Auditory verb learn test (AVLT), Digit Symbol Coding Test (DSCT), Clock Drawing Test (CDT), Verbal Fluency Test (VFT), Boston Naming Test (BNT) and Digit Span Test (DST).

For intergroup differences in neuropsychological scores, the one-way analyses of variance (ANOVA) test was used to compare continuous variables, and the χ^2^ test was applied to compare categorical variables.

### Data Preprocessing

The preprocessing steps were performed in DPARSF software.^1^ The functional images of each subject were slice-timing corrected, motion corrected, spatially normalized to the Montreal Neurological Institute (MNI) space template, resliced into 3×3×3mm^3^, spatially smoothed using a Gaussian kernel of a 6 mm full width at half maximum. Moreover, the images were further linearly detrended, filtered by a bandpass filter with 0.01–0.1 Hz, and 24 head motion parameters (Friston et al., 1996), white matter signal and CSF signal were regressed.

### Dynamic FC Estimation

The whole process of DFC analysis was performed using the sliding window approach (Allen et al., 2014). The 90 ROIs were generated based on the automated anatomical labeling (AAL) atlas. According to previous studies (Power et al., 2011; Cao et al., 2019; Long et al., 2019), the 90 ROIs were divided into nine groups: sensorimotor network (SMN), cingulo-opercular network (CON), auditory network (AUN), default mode network (DMN), visual network (VN), frontoparietal network (FPN), salience network (SN), subcortical network (SCN) and none. The ROI names of each group are listed in the Supplementary Table 1. The window length was set to 20 TR because previous studies pointed out that 30-60 s could be a reasonable choice of window length (Leonardi and Van De Ville, 2015; Preti et al., 2017). The rectangular window was shifted with a step of 1TR. For each subject, a total of 221 windows were produced. In each sliding window, the Pearson correlation of time courses of each pair of the 90 ROIs was calculated, and a 90×90 FC matrix was obtained.

### Clustering Analysis

The K-means algorithm was applied to the FC matrixes of all the subjects to identify the reoccurring FC patterns (Allen et al., 2014). According to the Akaike information criterion method (Burnham et al., 2002), the number of clusters was set to 7. The exemplars that consisted of windows with local maxima in FC variance along the time dimension were selected for each subject. The K-means algorithm was applied to the exemplars of all subjects and repeated 500 times with random initialization of centroids. After the resulting 7 cluster centroids were obtained, the K-means algorithm was applied to the FC matrixes of all the sliding windows of all the subjects by using the 7 cluster centroids as starting points. The final centroid of each cluster was regarded as the FC state.

### Dynamic FC Analysis

For each FC state of each subject, three DFC measures that included the mean dwell time, fraction of time and state transition probability were calculated. The mean dwell time is the average number of consecutive windows corresponding to each state. The fraction of time is the proportion of windows corresponding to each state. The state transition probability is the probability of switching from one state to another state. One-way ANOVA that used group as the between-subject factor were conducted to test intergroup differences for the mean dwell time, fraction of time and state transition probability of each state. Tukey’s honestly significant difference *post hoc* test was used to further compare the three groups (Abdi and Williams, 2010). All statistical analyses were carried out in SPSS 20.0.^2^

### Relationship Between Neuropsychological Assessment and Dynamic FC

We further investigated how DFC changes were relevant to the neuropsychological assessments. The analysis focused on the DFC measures that showed significant intergroup differences. The Pearson correlations between all the neuropsychological assessments in section “Neuropsychological assessment” and the DFC measures were calculated for the three groups separately.

### System Segregation

System segregation of each FC state was computed to qualitatively describing within-network correlations in relation to between-network correlations using the following formula (Chan et al., 2014; Bonkhoff et al., 2020):


$$\mathrm{System}\mathrm{segregation}=\frac{mean\left(Z_{w}\right)-mean\left(Z_{b}\right)}{mean\left(Z_{w}\right)}$$

where mean(*Z*_*w*_) represents the average of Fisher-transformed correlations within networks and mean(Z_b_) represents the average of Fisher-transformed correlations between networks. Note that we set all negative correlation values to zero. The segregation value was calculated for each sliding window of each subject. The system segregation of each FC state was computed by averaging segregations across the sliding windows corresponding to the state. One-way ANOVAs that used group as the between-subject factor were conducted to test intergroup differences for the system segregation of each state. The subjects that did not have a state was removed from the ANOVA test of the state. Tukey’s honestly significant difference *post hoc* tests were used to further compare the three groups.

The system segregations that showed significant intergroup differences were used to further investigate the relationship between segregation and all the neuropsychological assessments. The Pearson correlations between the segregations and the neuropsychological assessments were calculated in all the subjects of the three groups.

### Network-Based Statistical Analysis

The network-based statistic (NBS) method can yield substantially greater statistical power than generic methods to control the familywise error (FWE) (Zalesky et al., 2010). We applied the NBS method to compare the target FC state that showed significant intergroup differences in the mean dwell time/fraction of time and identify the connections that showed significant group differences. Considering that we had three groups in the experiment, the three groups were compared in pairs using the NBS method.

For each subject, Fisher’s r-to-z transformation was applied to the FC matrixes in all windows of the target FC state. Then, the mean FC matrix was obtained by averaging the FC matrixes across all the sliding windows for each target state. Two-sample *t*-tests were applied to each pairwise connectivity of the mean FC matrixes of any two groups to identify the intergroup difference. The t-statistic threshold was set to 3.2 to construct a set of suprathreshold links. For the suprathreshold links, we use a breadth-first search algorithm from the GRETNA toolbox^3^ to search connected components and calculate the size of each connected component.

For each two-sample *t*-test, a permutation test was performed to ascribe a p-value controlled for the FWE to each connected component based on its size. Each permutation test generated 1000 random permutations. For each permutation, all subjects were randomly reallocated into two groups, and the two-sample *t*-test was applied to the new two groups. The same t-statistic threshold (*t* > 3.2) was used to construct a set of suprathreshold links. The size of the maximal component in each permutation was obtained. For an observed component of size k, its p-value was measured as the total number of permutations where the size of the maximal component was greater than k divided by 1000. The components with *p* < 0.05 were selected as the suprathreshold links showing significant intergroup differences.

## Results

### Demography and Neuropsychological Assessment

Table 1 shows the demographic and clinical information of the three groups. No significant group differences among the three groups were found for age, gender or education. Most neuropsychological scores showed significant group effects.

**TABLE 1:** Demographic and clinical information.

	SIVD-aMCI (*n* = 34)	SIVD-naMCI (*n* = 30)	SIVD-Control (*n* = 37)	F/χ^2^	*p*
Age	67.41 ± 7.13	64.63 ± 6.73	66.14 ± 4.91	1.564	0.215
Male/female	21/13	15/15	20/17	0.939	0.625
Education, years	11.74 ± 3.52	11.07 ± 2.73	12.05 ± 3.16	0.821	0.443
MMSE	24.94 ± 3.35	26.60 ± 1.92	27.65 ± 1.53	11.447	< 0.001
ROCF-copy	30.03 ± 8.16	30.73 ± 6.76	33.59 ± 3.28	3.164	0.047
ROCF-recall	6.65 ± 6.00	12.76 ± 7.74	12.43 ± 5.32	9.934	< 0.001
TMT-A, s	77.73 ± 38.54	96.43 ± 40.15	56.00 ± 15.65	12.878	< 0.001
TMT-B, s	245.52 ± 101.87	264.30 ± 110.18	176.95 ± 58.05	8.766	< 0.001
SCWT-A, s	28.32 ± 8.24	28.13 ± 5.72	26.62 ± 7.58	0.573	0.566
SCWT-B, s	43.91 ± 14.31	38.17 ± 9.32	38.84 ± 12.46	2.144	0.123
SCWT-C, s	103.31 ± 39.72	99.97 ± 24.74	75.70 ± 19.53	9.387	< 0.001
AVLT N1-N5	12.33 ± 3.46	24.87 ± 5.79	31.35 ± 6.59	106.692	< 0.001
AVLT N5	0.64 ± 0.82	4.3 ± 1.39	6.30 ± 1.87	135.366	< 0.001
DSCT	23.97 ± 11.10	24.41 ± 7.86	35.57 ± 9.29	16.350	< 0.001
CDT	21.12 ± 5.98	23.83 ± 4.40	24.86 ± 3.06	6.141	0.003
VFT	33.97 ± 7.38	39.90 ± 8.52	46.19 ± 9.12	18.506	< 0.001
BNT	21.52 ± 3.87	22.41 ± 4.25	24.54 ± 3.22	6.006	0.003
DST	11.68 ± 2.41	12.21 ± 2.18	12.05 ± 1.91	0.512	0.061

*Values were expressed as mean ± standard deviation.* χ*^**2**^ test was applied in the comparison of sex. One-way analysis of variance was applied in the other comparisons.*

### Dynamic FC Analysis

**FIGURE 1:**
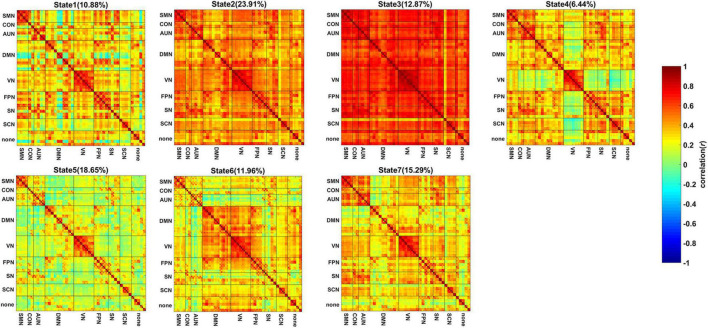
The centroid pattern of 7 FC states clustered by K-means. The percentage of occurrences is listed above each FC pattern. The FC pattern of each state represents Pearson correlation between each pair of brain regions.

The centroid patterns of the 7 FC states are shown in Figure 1. The ROI order of FC patterns in Figure 1 was the same as the Supplementary Table 1. All states showed strong positive connections within the SMN, VN and AUN. State 2, State 3, State 4, and State 7 showed strong positive connections between SMN and CON/AUN and between CON and AUN. Both State 2 and State 3 showed overall strong positive connections, while State 3 showed the strongest positive connections among the 7 states. For State 5, many connections were negative and close to zero. State 6 showed strong positive connections between DMN and VN and weak negative connections that were close to zero between the AUN and DMN/VN/FPN/SN/SCN. The connections between VN and FPN/SN/SCN in State 4 were negative and close to zero. State 1 showed weak negative connections close to zero between the parahippocampal/hippocampus gyrus in DMN and most other regions. State 7 showed weak negative connections close to zero between the medial prefrontal regions in DMN and most other regions.

**FIGURE 2:**
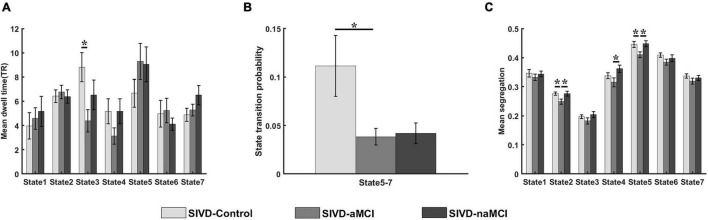
Measurements of dynamic FC for SIVD-Control, SIVD-aMCI and SIVD-naMCI. **(A)** The mean dwell time of 7 states. **(B)** The state transition probability that shows significant intergroup differences. **(C)** The mean system segregation of 7 states. *Represents *p* < 0.05.

Figures 2A,B show the comparison of the mean dwell time of the 7 states and the state transition probability of the states showing significant intergroup differences. One-way ANOVA revealed that the mean dwell time of the SIVD-Control group was significantly longer than that of the SIVD-aMCI group for State 3 [*F*_*group*_(2,98) = 4.100, Tukey corrected *p* = 0.014 < 0.05]. The state transition probability from State 5 to 7 of SIVD-Control was significantly greater than that of SIVD-aMCI [*F*_*group*_(2,98) = 3.914, Tukey corrected *p* = 0.039 < 0.05]. Moreover, the fraction of time did not show significant intergroup differences for the 7 states.

### Relationship Between Neuropsychological Assessment and Dynamic FC

**FIGURE 3:**
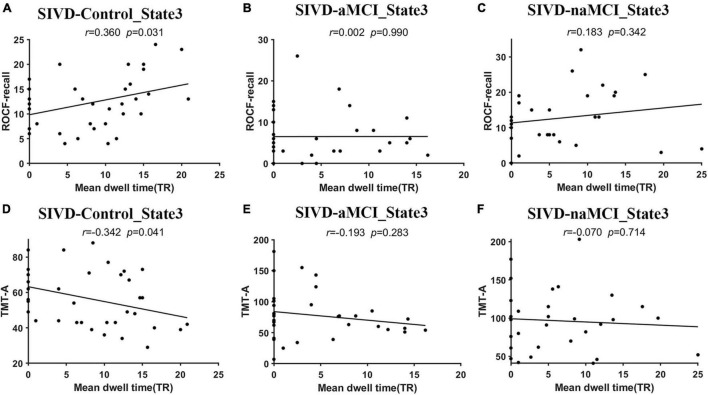
The relationship between the neuropsychological assessments and the measurements of dynamic FC. **(A–C)** The relationship between ROCF-recall and the mean dwell time of State 3 for SIVD-Control **(A)**, SIVD-aMCI **(B)** and SIVD-naMCI **(C)**. **(D–F)** The relationship between TMT-A and the mean dwell time of State 3 for SIVD-Control **(D)**, SIVD-aMCI **(E)** and SIVD-naMCI **(F)**.

Figure 3 displays the relationship that had a significant correlation between the neuropsychological assessments and the measurements of dynamic FC. For the SIVD-Control group, the mean dwell time of State 3 showed a significant positive correlation with ROCF-recall score (*r* = 0.360, *p* = 0.031 < 0.05) and a significant negative correlation with TMT-A score (*r* = −0.342, *p* = 0.041 < 0.05).

**FIGURE 4:**
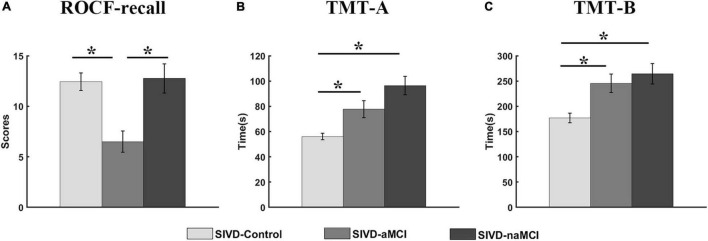
Neuropsychological assessments of ROCF-recall **(A)**, TMT-A **(B)** and TMT-B **(C)** for the three groups. *Represents *p* < 0.05.

The neuropsychological assessments that showed a significant correlation with dynamic FC are presented in Figure 4. For ROCF-recall, the average score of SIVD-aMCI was significantly lower than that of SIVD-Control [*F*_*group*_(2,95) = 9.934, Tukey corrected *p* = 0.001 < 0.05] and SIVD-naMCI [*F*_*group*_(2,95) = 9.934, Tukey corrected *p* = 0.001 < 0.05]. For TMT-A, the average time cost of SIVD-Control was significantly shorter than that of SIVD-aMCI [*F*_*group*_(2,97) = 12.878, Tukey corrected *p* = 0.018 < 0.05] and SIVD-naMCI [*F*_*group*_(2,97) = 12.878, Tukey corrected *p* < 0.001]. For TMT-B, the average time cost of SIVD-Control was significantly shorter than that of SIVD-aMCI [*F*_*group*_(2,95) = 8.766, Tukey corrected *p* = 0.007 < 0.05] and SIVD-naMCI [*F*_*group*_(2,95) = 8.766, Tukey corrected *p* = 0.001 < 0.05].

### System Segregation

**FIGURE 5:**
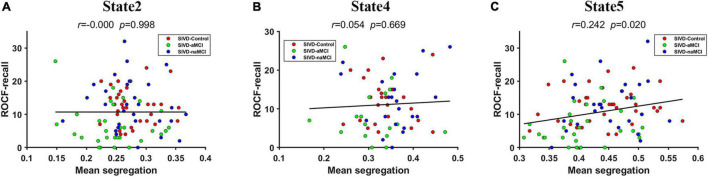
The relationship between the ROCF-recall score and the system segregations of State 2 **(A)**, State 4 **(B)** and State 5 **(C)**.

The mean system segregations of the 7 states are shown in Figure 2C. The SIVD-aMCI group showed decreased segregation than the other two groups for all the FC states. In contrast to SIVD-Control, SIVD-aMCI showed significantly decreased segregation for State 2 [*F*_*group*_(2,93) = 4.089, Tukey corrected *p* = 0.037 < 0.05] and State 5 [*F*_*group*_(2,92) = 4.209, Tukey corrected *p* = 0.040 < 0.05]. In contrast to SIVD-naMCI, SIVD-aMCI showed significantly decreased segregation for State 2 [*F*_*group*_(2,93) = 4.089, Tukey corrected *p* = 0.043 < 0.05], State 4 [*F*_*group*_(2,62) = 3.328, Tukey corrected *p* = 0.033 < 0.05] and State 5 [*F*_*group*_(2,93) = 4.209, Tukey corrected *p* = 0.034 < 0.05]. Among all the neuropsychological assessments, only the ROCF-recall scores showed significantly positive correlation with the segregation of State 5 (*r* = 0.242, *p* = 0.020 < 0.05). Figure 5 displays the relationships between the system segregation and the ROCF-recall score for State 2, State 4, and State 5.

### Network Connectivity Analysis

**FIGURE 6:**
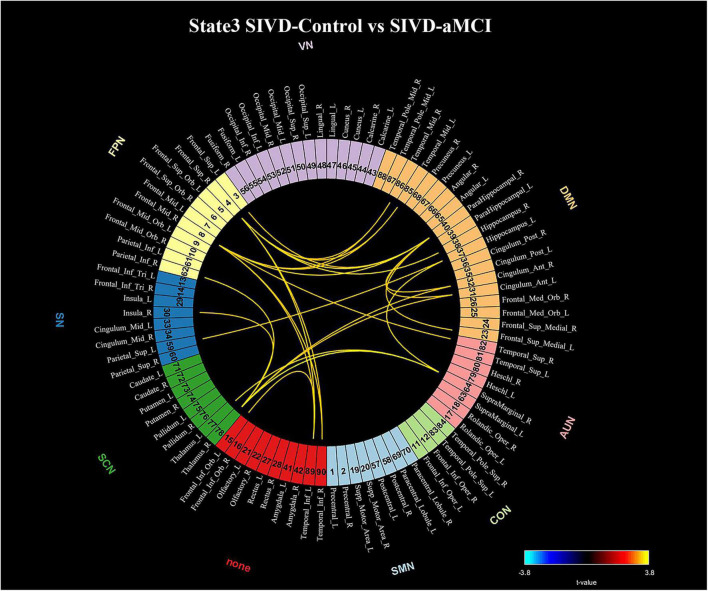
Significant connectivity differences in State 3 between the SIVD-Control group and SIVD-aMCI group for the NBS method. The numbers associated with the listed brain region are the indices in the AAL template.

The connections that showed significant intergroup differences in State 3 (*p* < 0.05) identified by the NBS method are displayed in Figure 6. The 25 connections that showed significant intergroup differences were observed between SIVD-Control and SIVD-aMCI. All 25 observed connections were significantly decreased in SIVD-aMCI compared with SIVD-Control. Among the 25 connections, most connections that included 8 DMN-FPN connections, 3 DMN-DMN connections, 2 DMN-SCN connections, 1 DMN-SN connection and 1 DMN-AUN connection occurred between the DMN and the other networks.

## Discussion

In this study, we explored the DFC differences among SIVD-Control, SIVD-aMCI and SIVD-aMCI and the relation between DFC and neuropsychological performance. Compared to SIVD-Control, SIVD-aMCI and SIVD-naMCI stayed less time in State 3 with overall strong connectivity strength, especially SIVD-aMCI and SIVD-Control showed significant difference in mean dwell time of State 3. The presence of aMCI and naMCI led to different degrees of reduction in the positive correlation between ROCF-recall and the mean dwell time of state 3 and a negative correlation between TMT-A and the mean dwell time of state 3. Moreover, the system segregation of State 5 with overall weak connections showed significantly positive correlation with ROCF-recall score. The memory impairment of SIVD-aMCI further reduced the system segregation of all the FC states. Our results verified the hypothesis that the presence of aMCI and naMCI resulted in different degrees of DFC changes and correlation changes between DFC and some neuropsychological performance.

### Dynamic FC Analysis

The dynamic FC analysis revealed 7 brain states reoccurring during rest. Both State 2 and State 3 showed stronger positive correlations for all the connections than the other states. Strong connections in the two states indicated that the brain regions were engaged in information transfer. Although the subjects were in the resting state, they could process some cognitive activities voluntarily (Chang and Glover, 2010; Allen et al., 2014). In contrast to State 2, the connections of State 3 were stronger, which may suggest that the subjects were engaged in various cognitive processes more actively and attentively in State 3. State 5 showed overall weaker connections than the other states. It was reported that the most frequent dynamic FC states during rest show attenuated between and within network connectivity (Allen et al., 2014; Nomi et al., 2017a,b; Denkova et al., 2019). Thus, weak connections in State 5 may suggest State 5 possibly was related to relaxation. For State 4, the connections between regions in VN and the regions in the other networks were negative and close to zero. When the subjects closed their eyes during blinking, the visual regions did not receive any visual input and did not need to interact with the other regions temporally. Thus, State 4 possibly was related to the state with eye closed. For State 1, the connections between the parahippocampal/hippocampus gyrus in DMN and the other regions were negative and almost close to zero. Because the parahippocampal gyrus and hippocampus gyrus in DMN were relevant to memory processing (Andrews-Hanna et al., 2014), the subjects possibly did not recall anything in State 1. For State 6, the regions in AUN were disconnected with the other regions, which may suggest that the subjects did not process auditory information in State 6. For State 7, the medial prefrontal regions in DMN showed weak connection with most regions. The medial prefrontal regions in DMN played a role in self-processing (Andrews-Hanna, 2012). State 7 possibly indicated that the subjects did not think much about themselves in this state.

Among the 7 states, State 3, with overall strong positive connections between and within brain regions/networks, showed significant intergroup differences in the mean dwell time. In contrast to SIVD-Control, both SIVD-MCI and SIVD-aMCI showed lower mean dwell time for State 3 (see Figure 2A), which was in line with the previous finding of a shorter dwell time of SIVD in the strongly connected DFC states compared to healthy controls (Fu et al., 2019). In particular, the differences between SIVD-Control and SIVD-aMCI were significant. The results indicated that the presence of aMCI and naMCI could cause SIVD patients to stay in State 3 for less time. Moreover, aMCI had a stronger impact on the mean dwell time of State 3 than naMCI. The overall strong connections of State 3 indicated that the brain regions possibly actively transferred information to perform various spontaneous cognitive processing in State 3. However, the cognitive impairment possibly impeded the information transition between regions (Seo et al., 2013; Sang et al., 2018). Therefore, the SIVD-MCI and SIVD-aMCI groups did not like to stay in State 3 with strong interactions between brain regions.

For the state transition probability, the transition probability from State 5 to 7 of the SIVD-Control group was greater than that of the SIVD-aMCI and SIVD-naMCI groups and the difference between SIVD-Control and SIVD-aMCI was significant (see Figure 2B). Among the 7 states, the percentage of occurrence of State 5 was the second highest. Moreover, State 5 showed overall weaker connections between and within brain regions/networks than the other states. The weaker connectivity in State 5 may indicate less efficient information transfer between and within brain regions/networks. Compared with State 5, State 7 showed relatively stronger positive connectivity between and within parts of brain regions/networks, which may suggest that the subjects produced more cognitive activities in State 7. The higher transition probability from State 5 to 7 of the SIVD-Control group may suggest that the SIVD-Control group was more likely to stay in the state with more cognitive activities. In contrast to the SIVD-Control group, the SIVD-naMCI and SIVD-aMCI groups showed a tendency toward a higher dwell time in State 5, although the intergroup difference was not significant (see Figure 2A). Thus, it can be inferred that the SIVD-naMCI and SIVD-aMCI groups were possibly more inclined to stay in State 5 with weak connectivity than the SIVD-Control group.

### Relationship Between Neuropsychological Assessment and Dynamic FC

The SIVD-Control group showed a significant positive correlation between the mean dwell time of State 3 and ROCF-recall scores, while the SIVD-naMCI and SIVD-aMCI did not show a significant correlation (see Figures 3A–C). For SIVD-Control, the positive correlation may indicate that State 3 possibly contributed to cognitive processing due to the strong interactions between brain regions in State 3. Due to impaired memory, SIVD-aMCI displayed significantly lower ROCF-recall scores than SIVD-Control and SIVD-naMCI (see Figure 4A). For SIVD-aMCI, the almost zero correlation between the dwell time and ROCF-recall score could be attributed to both reduced mean dwell time and reduced ROCF-recall scores. For SIVD-naMCI, the ROCF-recall score was not reduced, and the reduced positive correlation between dwell time and ROCF-recall scores was only attributed to the reduced mean dwell time of State 3.

For the TMT-A task, the SIVD-Control group showed a significant negative correlation between the mean dwell time of State 3 and the response time, while the SIVD-naMCI and SIVD-aMCI did not show a significant correlation (see Figures 3D–F). The results may suggest that State 3 was beneficial to the speed of performing the TMT tasks. For the SIVD-naMCI group, the impairment of executive function resulted in significantly increased response time of the TMT-A tasks compared with the SIVD-aMCI and SIVD-Control groups (see Figure 4B). Both the reduced mean dwell time and the increased response time of TMT tasks together resulted in the uncorrelation between the mean dwell time and response time for SIVD-naMCI. Moreover, the SIVD-aMCI group showed a significantly higher response time than the SIVD-Control group and a lower response time than the SIVD-naMCI group. The results may indicate that patients with memory impairment could also show impaired executive function to some extent (Zheng et al., 2012), although the impairment of executive function was not as severe as SIVD-naMCI. Thus, SIVD-aMCI showed only a reduced negative correlation, while SIVD-naMCI showed almost zero correlation. The correlation results further suggested that State 3 was a critical state for cognitive processing and that the cognitive impairment of SIVD could result in the DFC alteration of State 3. Moreover, the memory/execution function impairment of SIVD could largely reduce the correlation between the mean dwell time of State 3 and the performance of the ROCF-recall/TMT task for the SIVD-aMCI/SIVD-naMCI group.

Although State 3 was not dominant states among the 7 states, the results in this study suggest that the dwell time in State 3 was helpful for some cognitive processes. Because subjects in the resting state are not completely at idle state and can produce some spontaneous cognitive processing (Chang and Glover, 2010; Allen et al., 2014; Tavor et al., 2016; Zabelina and Andrews-Hanna, 2016; Kucyi et al., 2018), they can switch to some states that are relevant to cognitive processing and have stronger connectivity. However, cognitive impairment of SIVD might impede such a transition and cause patients to tend to stay in a state with weak connectivity.

### System Segregation

In contrast to SIVD-Control and SIVD-naMCI, SIVD-aMCI showed significantly smaller system segregation in State 2, State 4 and State 5. The segregation of SIVD-Control and SIVD-naMCI did not display significant differences for all the states. It was reported that both healthy and pathological ageing showed decreased segregation (Chan et al., 2014; Kim et al., 2017; Wig, 2017). Moreover, increased resting state network (RSN) modularity/segregation was positively correlated with improvement on the narrative task immediately post-therapy in the previous study (Duncan and Small, 2016), which may suggest reduced segregation to be a signature of impaired function. The results of neuropsychological assessments revealed that the SIVD-aMCI group showed predominant memory impairment as well as the execution function impairment to some extent (see Figure 4). Although the execution function of SIVD-naMCI was impaired, the system segregations of all the FC states were not reduced. The results could suggest that the reduced system segregation may have larger negative impact on memory than execution function. Thus, it could be the memory impairment of SIVD-aMCI that mainly resulted in the reduced system segregation of the FC states.

Among all the neuropsychological assessments, only the ROCF-recall scores showed significantly positive correlation with the system segregations of State 5, which was consistent with the previous finding that the segregation of association systems was significantly positive related to episodic memory (Chan et al., 2014). Individuals with higher ROCF-recall score produced higher system segregation of State 5. Compared to both SIVD-Control and SIVD-naMCI, SIVD-aMCI had significantly lower ROCF-recall scores (see Figure 4). Thus, SIVD-aMCI manifested significantly decreased segregation of State 5 than SIVD-Control and SIVD-naMCI. The results may suggest that the decreasing system segregation has negative consequences for behavior performance. Moreover, the observed relation between memory score and system segregation in this study provided further converging evidence to support that system segregation may be predictive of a summary measure of memory.

### Network Connectivity Analysis

The SIVD-aMCI group manifested significantly decreased functional connectivity that occurred mainly between the DMN and FPN in State 3 compared with the SIVD-Control group (see Figure 6), which was consistent with previous findings of reduced connectivity within/between the DMN, FPN and DAN (Sun et al., 2011; Yi et al., 2012; Schaefer et al., 2014; Zhou et al., 2015). For the SIVD-aMCI group, the reduced dwell time in State 3 with strong positive connectivity and less transition probability from State 5 with weak connectivity to State 7 could jointly contribute to the reduced functional connectivity of SIVD patients with cognitive impairment.

For the SIVD-aMCI group, the regions in FPN showed significantly reduced connections with parahippocampal cortex, hippocampus, inferior temporal cortex and middle temporal cortex. Both the parahippocampal cortex and hippocampus are important brain structures known to be involved in memory (Bohbot et al., 2015). The parahippocampal cortex is associated with episodic memory (Aminoff et al., 2013) and the hippocampus plays important roles in the consolidation of information from short-term memory to long-term memory, and in spatial memory that enables navigation (Buzsaki and Moser, 2013). The middle and inferior temporal gyri plays a key role in semantic memory (Chan et al., 2001) and FPN is involved in executive function and goal-oriented, cognitively demanding tasks (Uddin et al., 2019). For SIVD-aMCI, the reduced connectivities between FPN and the brain regions associated with memory in State 3 may suggest that the regions relevant to executive function did not interact with the regions relevant to memory actively during cognitive processing. Moreover, the previous study reported that gray matter reduction was observed in frontal cortex, the inferior temporal gyrus, the middle temporal gyrus and the parahippocampal gyrus of SVID (Zhou et al., 2016). As a result, SIVD-aMCI showed impairment in memory and executive function when they performed the ROCF-recall task and TMT task.

Moreover, SIVD-aMCI showed reduced connectivity between thalamus and insula in State 3. The gray volume reduction was also observed in both thalamus and insula of SIVD in the previous study (Zhou et al., 2016). The thalamus plays a crucial role in maintaining consciousness (Sherman and Guillery, 2002) and the insula is believed to be involved in consciousness (Craig, 2009). Due to the role of consciousness of both the thalamus and insula, the reduced connectivity between thalamus and insula of SIVD-aMCI may suggest it is harder for SIVD-aMCI to maintain consciousness and focus attention on cognitive activities than SIVD-control, which could lead to the worse performance in some neuropsychological assessments.

## Conclusion

This study investigated the impact of cognitive impairment (memory and execution function) on the DFC of SIVD patients. The results indicated that the cognitive impairment of SIVD mainly reduced the mean dwell time of State 3 with overall strong connections. The reduction degree of SIVD-aMCI was larger than that of SIVD-naMCI. Moreover, the memory/execution function impairment of SIVD changed the relationship between the mean dwell time of State 3 and the behavioral performance of the memory/execution task from significant to non-significant correlation. The memory impairment of SIVD-aMCI further resulted in the reduced system segregations of FC states, and the system segregation of State 5 showed significantly positive correlation with the memory score.

## Data Availability Statement

The raw data supporting the conclusions of this article will be made available by the authors, without undue reservation.

## Ethics Statement

The studies involving human participants were reviewed and approved by Institutional Review Board (IRB) of the State Key Laboratory of Cognitive Neuroscience and Learning in Beijing Normal University. The patients/participants provided their written informed consent to participate in this study.

## Author Contributions

LY and ZL conceptualized and designed the study. HS, HL, and JZ designed the experiment and collected the fMRI data. YX analyzed the data. YX and ZL wrote the manuscript. All authors contributed to the article and approved the submitted version.

## Conflict of Interest

The authors declare that the research was conducted in the absence of any commercial or financial relationships that could be construed as a potential conflict of interest.

## Publisher’s Note

All claims expressed in this article are solely those of the authors and do not necessarily represent those of their affiliated organizations, or those of the publisher, the editors and the reviewers. Any product that may be evaluated in this article, or claim that may be made by its manufacturer, is not guaranteed or endorsed by the publisher.

## Footnotes

1
http://rfmri.org/DPARSF

2
https://www.ibm.com/analytics/data-science/predictive-analytics/SPSS-statistical-software

3
https://www.nitrc.org/projects/gretna
