# Supplementary material for: Altered Dynamic Functional Connectivity in Subcortical Ischemic Vascular Disease With Cognitive Impairment
Source: Front Aging Neurosci. 2021 Dec 10;13:758137. doi: 10.3389/fnagi.2021.758137 (PMC8704998; doi:10.3389/fnagi.2021.758137)
Supplement: Supplementary file 1 [file Table_1.DOCX]

**Table 1** List of nine brain networks and their corresponding ROI defined in AAL.

| **Network** | **Regions** | **AAL index** |
| --- | --- | --- |
| **SMN** | **L precentral gyrus** | **1** |
|  | **R precentral gyrus** | **2** |
|  | **L supplementary motor area** | **19** |
|  | **R supplementary motor area** | **20** |
|  | **L postcentral gyrus** | **57** |
|  | **R postcentral gyrus** | **58** |
|  | **L paracentral lobule** | **69** |
|  | **R paracentral lobule** | **70** |
| **CON** | **L inferior frontal gyrus, opercular part** | **11** |
|  | **R inferior frontal gyrus, opercular part** | **12** |
|  | **L temporal pole: superior temporal gyrus** | **83** |
|  | **R temporal pole: superior temporal gyrus** | **84** |
| **AUN** | **L rolandic operculum** | **17** |
|  | **R rolandic operculum** | **18** |
|  | **L supramarginal gyrus** | **63** |
|  | **R supramarginal gyrus** | **64** |
|  | **L heschl gyrus** | **79** |
|  | **R heschl gyrus** | **80** |
|  | **L superior temporal gyrus** | **81** |
|  | **R superior temporal gyrus** | **82** |
| **DMN** | **L superior frontal gyrus, medial** | **23** |
|  | **R superior frontal gyrus, medial** | **24** |
|  | **L superior frontal gyrus, medial orbital** | **25** |
|  | **R superior frontal gyrus, medial orbital** | **26** |
|  | **L anterior cingulate and paracingulate gyri** | **31** |
|  | **R anterior cingulate and paracingulate gyri** | **32** |
|  | **L posterior cingulate gyrus** | **35** |
|  | **R posterior cingulate gyrus** | **36** |
|  | **L hippocampus** | **37** |
|  | **R hippocampus** | **38** |
|  | **L parahippocampal gyrus** | **39** |
|  | **R parahippocampal gyrus** | **40** |
|  | **L angular gyrus** | **65** |
|  | **R angular gyrus** | **66** |
|  | **L precuneus** | **67** |
|  | **R precuneus** | **68** |
|  | **L middle temporal gyrus** | **85** |
|  | **R middle temporal gyrus** | **86** |
|  | **L temporal pole: middle temporal gyrus** | **87** |
|  | **R temporal pole: middle temporal gyrus** | **88** |
|  | **L calcarine fissure and surrounding cortex** | **43** |
|  | **R calcarine fissure and surrounding cortex** | **44** |
| **VN** | **L cuneus** | **45** |
|  | **R cuneus** | **46** |
|  | **L lingual gyrus** | **47** |
|  | **R lingual gyrus** | **48** |
|  | **L superior occipital gyrus** | **49** |
|  | **R superior occipital gyrus** | **50** |
|  | **L middle occipital gyrus** | **51** |
|  | **R middle occipital gyrus** | **52** |
|  | **L inferior occipital gyrus** | **53** |
|  | **R inferior occipital gyrus** | **54** |
|  | **L fusiform gyrus** | **55** |
|  | **R fusiform gyrus** | **56** |
| **FPN** | **L superior frontal gyrus, dorsolateral** | **3** |
|  | **R superior frontal gyrus, dorsolateral** | **4** |
|  | **L superior frontal gyrus, orbital part** | **5** |
|  | **R superior frontal gyrus, orbital part** | **6** |
|  | **L middle frontal gyrus** | **7** |
|  | **R middle frontal gyrus** | **8** |
|  | **L middle frontal gyrus, orbital part** | **9** |
|  | **R middle frontal gyrus, orbital part** | **10** |
|  | **L inferior parietal, but supramarginal and angular gyri** | **61** |
|  | **R inferior parietal, but supramarginal and angular gyri** | **62** |
| **SN** | **L inferior frontal gyrus, triangular part** | **13** |
|  | **R inferior frontal gyrus, triangular part** | **14** |
|  | **L insula** | **29** |
|  | **R insula** | **30** |
|  | **L median cingulate and paracingulate gyri** | **33** |
|  | **R median cingulate and paracingulate gyri** | **34** |
|  | **L superior parietal gyrus** | **59** |
|  | **R superior parietal gyrus** | **60** |
| **SCN** | **L caudate nucleus** | **71** |
|  | **R caudate nucleus** | **72** |
|  | **L lenticular nucleus, putamen** | **73** |
|  | **R lenticular nucleus, putamen** | **74** |
|  | **L lenticular nucleus, pallidum** | **75** |
|  | **R lenticular nucleus, pallidum** | **76** |
|  | **L thalamus** | **77** |
|  | **R thalamus** | **78** |
|  | **L inferior frontal gyrus, orbital part** | **15** |
|  | **R inferior frontal gyrus, orbital part** | **16** |
|  | **L olfactory cortex** | **21** |
| **none** | **R olfactory cortex** | **22** |
|  | **L gyrus rectus** | **27** |
|  | **R gyrus rectus** | **28** |
|  | **L amygdala** | **41** |
|  | **R amygdala** | **42** |
|  | **L inferior temporal gyrus** | **89** |
|  | **R inferior temporal gyrus** | **90** |

Abbreviations: L, left; R, right; sensorimotor network, SMN; cingulo-opercular network, CON; auditory network, AUN; default mode network, DMN; visual network, VN; frontoparietal network, FPN; salience network, SN; subcortical network, SCN.
